# Public engagement in Malawi through a health-talk radio programme ‘*Umoyo nkukambirana*’: A mixed-methods evaluation

**DOI:** 10.1177/0963662516656110

**Published:** 2016-06-30

**Authors:** Deborah Nyirenda, Tamara Chipasula Makawa, Greyson Chapita, Chisomo Mdalla, Mzati Nkolokosa, Thomasena O’byrne, Robert Heyderman, Nicola Desmond

**Affiliations:** Malawi Liverpool Wellcome Trust Clinical Research Programme, Malawi; University of Malawi-College of Medicine, Malawi; Liverpool School of Tropical Medicine, UK; Malawi Liverpool Wellcome Trust Clinical Research Programme, Malawi; Malawi Broadcasting Corporation, Malawi; Malawi Liverpool Wellcome Trust Clinical Research Programme, Malawi; Malawi Liverpool Wellcome Trust Clinical Research Programme, Malawi; University College London, UK; Malawi Liverpool Wellcome Trust Clinical Research Programme, Malawi; Liverpool School of Tropical Medicine, UK

**Keywords:** community engagement, evaluation, Malawi, medical research, public engagement, radio listening club, radio programme

## Abstract

Radio is an effective source of health information in many resource poor countries. In Malawi, 53% of households own radios however few radio programmes in Malawi focus on health issues in the context of medical research. An interactive health-talk radio programme ‘*Umoyo nkukambirana*’ was introduced by Malawi-Liverpool-Wellcome Trust Clinical Research Programme on a national radio station. The aim was to increase awareness of health and medical research, and improve engagement between researchers, healthcare workers and the public. The content and presentation were developed through participatory community consultations. Focus Group Discussions were conducted with established Radio Listening Clubs whilst quantitative data was collected using toll free FrontlineSMS to explore national response. A total of 277 to 695 SMS (Median: 477) were received per theme. The majority of SMS were received from men (64%) and mainly from rural areas (54%). The programme improved knowledge of medical research, health and dispelled misconceptions. This study suggests that the radio may be an effective means of increasing the exposure of men to health information in resource poor settings.

## 1. Introduction

In recent years, public and community engagement have been increasingly recognised as a critical component of international medical research (Dickert and Sugarman, 2005; National Co-ordinating Centre for Public Engagement, 2010). The UK National Co-ordinating Centre for Public Engagement (2010) defines public engagement as ‘the myriad of ways in which activity and benefits of higher education can be shared with the public. Engagement is by definition a two way process involving interaction and listening, with the goal of generating mutual benefit’. There are different approaches to engaging with the public including stakeholder workshops, exhibitions, debates and citizens’ jury; however, these often target a specific audience and are confined to a particular location. Television and social media have been utilised to engage with the public in developed world contexts (Anikeeva and Bywood, 2013; Korda and Itani, 2013); however, these may be less effective in resource-poor settings where they are primarily accessible to a smaller proportion of the population. In contrast, the radio is an effective source of information in resource-poor contexts due to increased accessibility and the potential for wide coverage compared to other forms of media (Lunt, 2005). This potential to reach wider audiences, including less literate populations, derives from the portability and affordability of radio.

A number of studies evaluating the impact of radio interventions on health have clearly demonstrated its potential to improve knowledge and attitudes towards child feeding practices, tuberculosis (TB) and maternal health (Monterrosa et al., 2013; Onyeonoro et al., 2013; Radoff et al., 2013). The increasing emphasis on public engagement in research necessitates the need for evidence-based practices; however, there are challenges to assess the impact of public engagement due to lack of consensus on the meaning of public engagement, variations in practice, contexts and research participants (Milton et al., 2011; Sieber, 2010; Tindana et al., 2011). Studies have shown that participation in dialogue has a positive effect on participant attitudes towards the topic of discussion (Zorn et al., 2012). Few studies have, however, presented public engagement projects in practice (Marsh et al., 2008, 2010) outcomes of public engagement (South and Phillips, 2014) or using radio as a tool to improve public engagement with health research. Drawing on this background, the Malawi Liverpool Wellcome Trust Clinical Research Programme (MLW), based at the University of Malawi College of Medicine, introduced a health-talk radio programme to improve engagement between researchers, clinicians and the general public on Malawi Broadcasting Corporation (MBC) radio. This article will discuss the evaluation, focusing on national responses as well as impact on knowledge and reported treatment-seeking behaviour.

## 2. Setting

Malawi is located in Southern Africa with a population of 17,215,000, the majority of whom (84%) reside in rural areas (The World Bank, 2015). Malawi is ranked 173/187 in the Human Development Index 72% of the population live on less than US$1.25 a day (UNDP, 2015). Quality of education remains inadequate due to shortage of teachers, education materials and infrastructure investment (Chimombo, 2005). Consequently, literacy levels are comparatively low; 67% of women and 81% of men are literate (National Statistics Office (NSO) & ICF Macro, 2011) translating to poor knowledge of health issues and the potential for inappropriate health-seeking choices (Desmond et al., 2013). Access to media is also limited with 53% of households owning radio and only 11% possessing a television (NSO & ICF Macro, 2011). A total of 41% of the population own mobile phones (NSO & ICF Macro, 2011) with a geographical coverage of 80% across the country (GSMA, 2014).

Malawi has a high disease burden evidenced by a crude death rate of 12% (UNICEF, 2014). This is largely due to infectious diseases such as HIV/AIDS, malaria, pneumonia, diarrhoea and tuberculosis (TB) (WHO, 2015) with an increasing epidemic of non-communicable disease as in other Sub-Saharan Africa (SSA) settings (Amuyunzu-Nyamongo et al., 2013; SanJoaquin et al., 2013). Relative to population, Malawi produces high numbers of research outputs through both local and international institutions (WHO, 2014), but there remain common misconceptions about health research within the country which affect implementation of research (Mfutso-Bengo et al., 2008a, 2008b). This necessitates the need to improve understanding of health research and engagement between researchers and the general public. Media coverage on health issues is low, erratic and, in some cases, inaccurate (African Woman and Child Feature Services, n.d.; Eldis, 2004). Most of the broadcasting houses in Malawi allocate a lot of airtime to sports and entertainment (up to 74%), while health programmes are allocated 1%–17% of airtime only (MACRA, 2015). Given this, research findings are rarely adequately reported nationally and dissemination relies on national and international conferences as well as peer-reviewed scientific journals and newspapers. These forums are, however, inaccessible to those whose needs the research is designed to benefit.

### *Umoyo Nkukambirana* radio programme

The *Umoyo Nkukambirana* (literally translated as ‘let’s talk about health’) radio programme sits within an active public engagement programme at MLW led by the Science Communication department. MLW is one of the six Wellcome Trust–funded major overseas programmes conducting cutting edge global health research. The radio programme was developed to increase awareness of health and medical research and to improve engagement between researchers and clinicians with the media and general public. The radio programme was produced in collaboration with the Development Broadcasting Unit (DBU) of MBC whose main role is to use participatory communication approaches to promote national dialogue around development and health issues. DBU works with rural communities by organising radio listening clubs (RLCs) that meet regularly to listen to recorded programmes for feedback. In addition, RLCs also identify problems in their community and discuss with relevant service providers to develop a locally relevant action plan. During the establishment of the MLW radio programme, the Science Communication department worked with six pre-existing RLCs, previously established through DBU. The RLCs were from six districts, namely, Balaka, Blantyre, Chiradzulu, Dedza, Mwanza and Ntcheu, where Chichewa is the main language.

A participatory radio broadcasting approach was adopted to ensure relevance and ownership (Manyozo, 2007) by involving the RLC in generating content for the programme through local case studies, poetry, songs, questions and drama. Prior to implementation, researchers from MLW, journalists and editors from various media houses, District Executive Committee members and RLC members were consulted to inform the development of content and presentation of the radio programme. Topics featured during the pilot phase were identified through this consultation process, and each was allocated four or five programmes in 1 month of broadcasting to ensure adequate coverage. Topics considered most salient were research and blood, diabetes, TB, drugs and vaccines, cancer, malaria, meningitis and DNA. The radio programme was advertised widely through the radio, newspaper, posters and through community launch events. Medical researchers presented health topics in the local language, Chichewa, and responded to questions asked by listeners through SMS and via the RLC. Every week, the presenter asked a question relating to the weekly topic. Five lucky listeners who responded correctly to questions won t-shirts. This was done to encourage listeners’ participation in the programme. The pilot programme was aired every Sunday for 30 minutes from 5:00 p.m. to 5:30 p.m. on MBC Radio 1, a station that has the highest geographical coverage across all districts in Malawi. The programme was piloted for 9 months from August 2012 to April 2013.

## 3. Evaluation methods

An integrated monitoring and evaluation approach using mixed methods was designed to assess process and impact of the radio programme. The aim of this evaluation was to assess and solicit feedback from national and more localised responses to improve programme relevance, optimise content and understand impact. Focus group discussions (FGDs) were conducted with RLC, and the national response was monitored quantitatively through FrontlineSMS (www.frontlinesms.com).

## 4. Focus Group Discussions (FGDs) with Radio Listening Clubs (RLCs)

A topic guide with 13 themes was developed including preferred sources of information on health, programme impact on knowledge, attitudes, behaviour and feedback on content and presentation. Convenience sampling was used to select RLC from six districts already working with MBC (DBU) to participate in 10 FGDs.The RLC comprised community representatives including men, women and youth who were trained to raise development issues within their communities with the relevant authorities. Each FGD lasted between 50 and 90 minutes and was facilitated in the local language by a trained social scientist (D.N.) and the head of the Science Communication programme (T.C.M.). FGDs were recorded using a digital recorder, and these recordings were transcribed and translated to English by experienced transcribers and translators based at MLW.

## 5. Monitoring national response

Quantitative monitoring data were collected weekly through FrontlineSMS to explore the national response to the programme. Towards the end of each programme, the medical researcher posed a question to the audience related to the weekly topic. Listeners were frequently requested to send toll-free SMS in response to the question or to ask additional questions and provide feedback. Listeners were encouraged to provide socio-demographic details of their location, age and gender to assess the national response to the radio programme. SMS senders were entered into a draw with the opportunity of winning a t-shirt.

## 6. Data analysis

Qualitative data from FGD were coded manually and analysed using a combination of thematic content and framework analysis. Transcripts were read and re-read to provide a general overview of the main findings. An initial coding framework was developed by two social scientists (D.N. and N.D.) based on a combination of the research questions and more inductively through emerging themes from the data. A matrix was developed in Microsoft Excel using the sub-themes as a framework and populated with findings from each FGD. Recurring themes were identified and further grouped into the core themes of accessibility, knowledge and impact on treatment seeking. These three main themes were then explored and triangulated across individual FGD, and a data summary was produced for each. In cases where anomalies were identified in the SMS data such as gender differences in response to the radio programme, the lead researcher returned to the field to conduct follow-up discussions with RLC members to understand why response was higher among men compared to women. Socio-demographic details and text content were analysed to explore national response to topics within the radio programme. Details of SMS senders were entered into SPSS version 16 (IBM) against topics. Descriptive statistics were produced to explore geographic location, age and sex differentials by topic, including responses to weekly questions and free comments and questions. These latter were grouped into themes to complement the qualitative findings. Once definitive findings had been reached, representative quotes for each sub-theme were identified from FGD transcripts and text content.

## 7. Results

### Reach and accessibility of the programme

Most participants felt that the duration of the programme (30 minutes) was very short and insufficient to develop themes adequately: ‘The (radio) programme is too short, when you get a phone to ask a question (through SMS) you just realise that the programme has come to an end’ (Woman, FGD with RLC A). The starting time for the programme (5:00 p.m.) was noted as inconvenient, especially for women, since this was a time when they were tasked with household responsibilities. In addition, most people who did not have radios, particularly women, indicated that they had challenges to seek permission from spouses to go and listen to the radio programme in other people’s homes because it was dark:

The radio programme is good and we are learning a lot from doctors … however the starting time for the programme is not good because it is late and most women are busy with household work in the kitchen … it is also hard for us to go and meet with others and listen to the radio programme. (Woman, FGD with RLC B)

**Figure 1.**
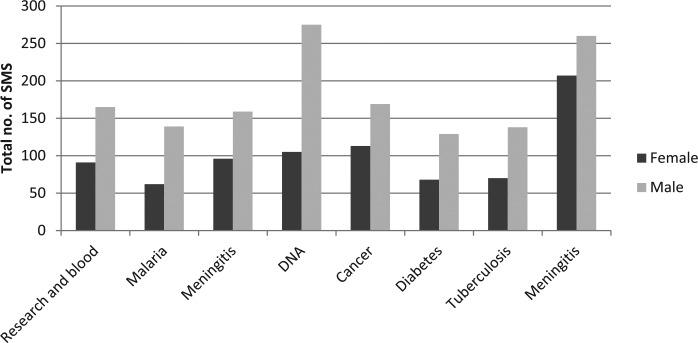
National response by gender.

There were mixed perspectives on the ideal timeslot for the programme; RLC from rural areas preferred the programme to be aired in the early afternoon, while RLC from urban areas wanted the programme to be aired in the late afternoon. This reflects contrasting social environments experienced by RLC members; women in rural areas were able to walk to attend RLC meetings and listen to the programme as a collective. Women in urban areas, however, preferred the programme to be aired in the evening so that they could listen in the comfort of their homes. The majority of men and women felt that coverage would be improved through repeat broadcasts or using other geographically targeted, radio stations. Responses to the programme through SMS were limited due to issues around access to phones as reflected in Demographic and Health Survey (DHS) findings that only 41% of the population own mobile phones (NSO & ICF Macro, 2011). FGD participants also preferred phone calls over SMS due to challenges to send SMS which also reflects low levels of literacy among listeners. A total of 277–695 SMS (median: 477, mean: 448) were received by monthly theme (Table 1). SMS senders were aged between 8 and 76 years (median: 27 years). More SMS were sent by men (64%) compared to women across all themes (see Figure 1) reflecting gender differences observed in the Malawi DHS 2010 (NSO & ICF Macro, 2011). Text messages were received from residents in all 28 districts of Malawi although the response was higher in the southern region (1429) followed by central (1119) and northern regions (304) reflecting population density in each region. In addition, presenting the programme in Chichewa may have presented the risk of excluding other people from participating since Malawi is a multilingual society. SMS data also reflected the urban–rural differences identified during the FGDs. A total of 46% of SMS came from four urban centres: Lilongwe (22%), Blantyre (17%), Zomba (5%) and Mzuzu city (2%) in comparison with the remaining 24 districts (54%), and this again reflects access to mobile phones (GSMA, 2014).

**Table 1.** National response by age.

Monthly theme	Total no. of SMS	Age (years)				
		<15	16–25	26–35	36–45	>46
Meningitis	695 (19%)	4	246	143	84	11
DNA	595 (17%)	1	49	100	20	6
Meningitis	483 (13%)	0	46	40	23	9
Research and blood	477 (13%)	3	50	61	26	15
Cancer	382 (11%)	1	73	98	20	8
Tuberculosis	361 (10%)	7	85	67	27	19
Malaria	317 (9%)	1	36	39	18	33
Diabetes	277 (8%)	0	72	43	12	9
Total		17	657	591	230	110

## 8. Knowledge of health research

During initial consultation meetings, RLC members demonstrated a lack of understanding of the rationale underlying medical research and exposure to research findings. RLC members described medical research as ‘sanitation’, ‘changes in medicine’, ‘work done by community based health workers’ and ‘assessing the availability of pit latrines in a community’; only a few participants described it as ‘a way of finding solutions to health problems in an identified area’.

In response, the Science Communication group introduced the programme with the first topic of ‘research and blood’ to introduce people to the practice of medical research. Following implementation, FGD with RLC demonstrated an improved understanding of medical research and its role when introducing new drugs and interventions within these groups:

This programme has enlightened us in the villages, we just heard that Fansidar (Sulphadoxine and Pyrimethamine) phased out and they have introduced LA (Lumefantrine Artemether) … we did not know why Fansidar was removed but now we are aware that research was done before they introduced LA. (Woman, FGD with RLC C)

## 9. Demystifying misconceptions and increasing knowledge

Listeners and FGD members stated that the radio programme increased their understanding of symptoms, causes and prevention of a range of diseases. Table 2 demonstrates these initial knowledge gaps by detailing a selection of questions asked through SMS. The radio programme was perceived to clear up misconceptions about malaria, meningitis, DNA, diabetes and research. Myths about cancer were also dispelled through exposure to the programme, but concerns were frequently expressed over difficulties in understanding the topic of DNA due to challenges in translating scientific terms into a locally appropriate language.

**Table 2.** Examples of weekly questions through SMS from radio listeners.

Monthly theme	Question	Date
Research and blood	Do you wait for a person to become really sick to conduct research?	5 August
	Do you conduct research on anyone?	5 August
	Can a person who is HIV negative but from a discordant couple donate blood?	19 August
Malaria	How does this disease (malaria) start? Is it food, poor domestic hygiene, how can we prevent it?	23 September
	What is the difference between meningitis and malaria? Because these two affect people at the same time	23 September
Meningitis	They say meningitis is associated with TB, is it true?	7 October
	What is the connection between this disease (meningitis) and malaria? Because some of us don’t know how to differentiate them	7 October
DNA	Is there any difference between DNA and blood cells? How different is DNA from genes? Thank you for discussing DNA.	4 November
	If the body runs out of DNA, are there some drugs that can replace DNA in the body?	11 November
	Where do you find DNA in the body? Does it mean that people who are infertile lack DNA?	11 November
	The programme is good because it is enlightening us about science	25 November
Breast cancer	If a woman was diagnosed with breast cancer while breastfeeding, can she continue to breastfeed? Will the child get infected too?	9 December
Diabetes	What problems can one experience if they have got low sugar?	
	Does diabetes make people urinate frequently? And does it make one become mentally disabled?	27 January
Tuberculosis	How long does TB [Tuberculosis] take in order to be visible? And can you go to the hospital to check if you have TB?	10 January

Participants stated that before the programme, they used to treat any fever with malaria drugs: ‘We used to associate fever with malaria and could not understand why doctors (clinicians) would tell us that we do not have malaria when we have fever’ (Man, FGD with RLC E). Malaria was also seen as being caused by overworking and getting soaked during the rainy season: ‘We used to believe that if you get soaked in the rains you suffer from malaria, but we have now learnt that malaria is caused by mosquitoes’ (Woman, FGD with RLC D).

Symptoms of other diseases such as stiff neck and convulsions were often associated with witchcraft, and this interpretation commonly prompted people to seek traditional medicine and delay presentation for appropriate biomedical treatment: ‘we used to associate convulsions with witchcraft and we would consult traditional healers for medicine, we have now learnt that it could be due to malaria’ (Woman, FGD with RLC C). Some FGD participants stated that they had not understood why blood is drawn for donation or laboratory tests, associating this with blood stealing and Satanism; these beliefs were addressed and new understanding reached on reasons for drawing blood.

For example, a male participant during a FGD admitted, ‘We were scared to donate blood; we were wondering what they want the blood for? And where do they take it to? But we are now aware that it is meant to be used in cases of emergencies’ (Man, FGD with RLC A). In general and tellingly, people appreciated the facilitative role the programme played in increasing access to medical professionals. The radio programme also enabled them to pose questions they were often unable to pose during regular engagement through the health service as illustrated by a male participant: ‘The programme has given us an opportunity to talk to doctors who are specialists … we are able to know about dangers of various illnesses and whether they are treatable or not’ (Man, FGD with RLC C).

Overall, the monthly themes of meningitis and DNA attracted the highest numbers of SMS (19% and 16%, respectively), while the theme of diabetes received the lowest (8%). Findings from FGDs indicated that response was higher for meningitis and DNA because these themes were rarely discussed elsewhere, either on the radio, during health talks at clinics, or in community sensitisation and health promotion meetings. FGD participants did not explain why response was low on the theme of diabetes, but this may reflect a perception that diabetes is a disease of the affluent or the west (Msyamboza et al., 2014).

In order to find out whether the featured radio programmes were sufficiently understood by the public, we conducted an assessment of correct responses to each weekly question related to the topic of the programme. The questions were formulated in collaboration between the researcher and presenter based on the content of discussions. A list of all weekly questions has been included in Supplementary Appendix (pus.sagepub.com). The majority of SMS responses to these questions were correct across most themes: DNA (93%), malaria (89%), research and blood (87%) and meningitis (85%), suggesting active and engaged listening. Correct SMS responses were lower for the cancer theme (57%), reflecting its complexity, particularly among women.

## 10. Impact on reported or intended treatment-seeking behaviour

Rigorous evaluation of impact on treatment seeking was not possible given the design of the evaluation framework. The researchers were, however, able to explore reported or intended behaviour that FGD participants associated with exposure to the radio programme. Participants from some RLCs stated that knowledge acquired through the radio programme empowered them to improve their own responses to illness experienced within the household. Most people claimed to take over-the-counter painkillers or traditional medicines when they feel sick, primarily due to long walking distances, shortage of drugs and protracted waiting times at health facilities. One female participant stated, ‘In the past, we would delay to take a sick child to hospital by giving them painkillers at home but now we are aware that we need to rush to hospitals for diagnosis’ (Woman, FGD with RLC A). Claims that response to illness was improved focused particularly on meningitis programmes that included case studies. The programmes featured a young man who recovered from meningitis after presenting promptly to hospital and receiving medical treatment. Another radio programme featured a young girl who suffered severe disability due to delays in diagnosis and treatment caused by initial misinterpretation of the disease. Participants claimed to have been affected by this story and that this motivated them to seek prompt biomedical treatment to ensure proper diagnosis rather than giving home-based medication. For example, one female participant stated,

Previously, when we have neck pains we would get traditional medicine to put around the neck … I had severe neck pains that I couldn’t turn my head left or right and it was after they had a programme on this disease that stiffens the neck (meningitis) … I realised that the symptoms I had were similar to the ones mentioned in the radio programme and I rushed to the hospital where they gave me medication and I am now well … If I had gone to the traditional healer I would have delayed. (Woman, FGD with RLC C)

Knowledge gained through listening to the radio programme was also felt to empower some individuals to counsel and encourage others to seek medical treatment on time, spreading messages within communities beyond those who listened to the programme.

## 11. Discussion

This study reports experiences and outcomes from implementing a radio programme as part of broader public engagement activities at a large international research institution in Malawi. The radio programme promoted dialogue between researchers and the public by providing a platform for medical researchers to reach out to listeners. Most importantly, listeners were also able to ask questions they felt unable to pose during treatment-seeking encounters with health professionals.

While previous studies have demonstrated the range of misconceptions about health research in resource-poor settings (Marsh et al., 2008; Mfutso-Bengo et al., 2008a, 2008b). Findings from this study demonstrate how the radio can be used as a tool to increase public understanding, demystify research and clear up misconceptions. The radio programme generated interest throughout Malawi, facilitating access to health information among people from all districts and answered to the high demand for interactive programmes about health, with regular requests for increased transmission times and rebroadcasts.

The use of quantitative research alongside qualitative research enriched the findings by providing insights and explanations for quantitative findings. In particular, we noted gender differences in responses to the programme at a national level with higher number of responses from men and urban areas. This is in keeping with findings from Malawi (NSO & ICF Macro, 2011) and elsewhere (Meekers et al., 2007; Onyeonoro et al., 2013) demonstrating that literacy levels, cell phone and radio ownership is higher among men and in urban areas. Greater response rates from men to the health-talk radio programme should be understood within a broader understanding of men’s engagement with health and health services.

Low participation of men in health interventions or research has been widely reported (Manda-Taylor, 2013). (Morfaw et al., 2013) In light of this, findings from this study suggest that the radio may be an effective means of exposing men to health information in resource-poor settings due to their increased access to radio. This is likely to be beneficial in settings where men are often household decision-makers (Manda-Taylor, 2013), and their understanding of research and health is likely to affect household decisions to seek biomedical treatment or to enrol or withdraw from research studies.

The MLW health-talk programme was developed to increase awareness among both men and women, and thus, identification of an appropriate transmission time for the programme is important. In this case, the situation analysis informing the development of the pilot was found to be inconsistent with women’s needs, particularly for rural women, engaged in domestic chores. The lower response rate from women is likely to have been a reflection of phone access, ownership and literacy as well as time to listen and respond to the programme. Women also stated that they failed to engage as they felt they lacked knowledge to respond to the weekly questions through SMS. Careful consideration of the respective needs of men and women should, therefore, be included in the design of programme content and process to improve knowledge of health issues.

The increased response to the meningitis programmes where case studies featured more heavily suggests the need to develop programmes that focus on increasing knowledge through personal examples since these are likely to sustain the empathetic interest of listeners. This is in line with another study that has shown that sharing personal experiences about the safety of vaccines promoted immunisation (Kempe et al., 2011). While another study has demonstrated that provision of information to correct myths about vaccines was not effective to promote immunisation (Nyhan and Reifler, 2015), these findings suggest that personal examples of experiences with an illness were effective to improve response to illnesses.

Strength of this programme lies in the participatory development of the themes. Prior to implementation, consultation meetings were conducted with relevant stakeholders to determine content and presentation. Communities were also engaged through involvement of RLCs in the production of radio through panel discussions, drama, poetry, personal stories and songs. This ensured that content and presentation were culturally appropriate, compelling and relevant to the lives of the listeners. Featuring songs in a health intervention was seen as effective to promote behaviour change in Gambia (Panter-Brick et al., 2006), and our findings suggest that personal stories can potentially influence positive behaviour change. Presentation in the local language (Chichewa) ensured access and engagement among those with lower levels of education and less exposure to the adopted national language, English. However, this may have presented challenges to non-Chichewa speakers to understand or participate in the programme. FGDs were conducted in districts where Chichewa was the predominant language and thereby excluding views of non-Chichewa speakers.

Inevitably, any attempt to evaluate an initiative of this kind faces methodological and practical limitations relating to measuring levels of engagement, empowerment of listeners and impact more broadly. Challenges of measuring effects and outcomes of public engagement have also been widely acknowledged (South and Phillips, 2014; Wellcome Trust, n.d.). Measuring the extent of listenership through reliance on SMS response to programmes is limited since it reflects a number of factors that cannot easily be measured during the analysis such as differences in access to radios and phones. Consequently, the total number of SMS collected is not representative of all listeners. In addition, challenges to the system included an unreliable network and erratic electricity supplies. Data analysis of SMS response was also limited due to failure of respondents to include socio-demographic details such as age, gender and location, despite a prize draw to emphasise this need and promote inclusion of these data.

Additionally, engaging RLC in FGD had its own challenges due to previous engagements with DBU and other organisations which gave an impression that their role was to give feedback on the radio programme. The authors also acknowledge that findings reported in FGD on treatment seeking may have been subject to reporting bias because behaviour change is complex and depends on various personal and social factors to sustain the desired change (Kaufman et al., 2014; Panter-Brick et al., 2006). As such, this study cannot ascertain the effects on treatment-seeking behaviour, taking into account the cross-sectional nature of the study and absence of a comparison group. However, despite these methodological limitations, the findings of this evaluation provide an important contribution to the field of community engagement practice and evaluation. These findings will also inform the future implementation of the MLW radio programme and its evaluation.

## 12. Conclusion

Our research findings have demonstrated that radio provides an effective means of public engagement in resource-poor settings because it increases access to information particularly for men and hard to reach populations with low levels of literacy. Throughout the development of the radio programme, the Science Communication team worked closely in consultation with communities. Integrated evaluation approaches were designed to gather feedback for programme improvement and assess its impact on community knowledge and response to illness. We conclude that health-talk radio programmes have the potential to improve knowledge about health, medical research and improve presentation at health-care providers if the content is developed in response to listeners’ needs. Other evaluation strategies such as pre- and post-intervention surveys or randomised controlled trials in Burkina Faso (DMI, 2014) are likely to be effective in assessing reach and particularly in exploring long-term effects on improving health and social outcomes.
